# Glucosylation of T-2 and HT-2 toxins using biotransformation and chemical synthesis: Preparation, stereochemistry, and stability

**DOI:** 10.1007/s12550-018-0310-9

**Published:** 2018-03-06

**Authors:** Henning Sören Schmidt, Mareike Schulz, Christine Focke, Stefanie Becker, Benedikt Cramer, Hans-Ulrich Humpf

**Affiliations:** Institute of Food Chemistry, Westfälische Wilhelms-Universität Münster, Corrensstrasse 45, 48149 Münster, Germany

**Keywords:** Modified mycotoxins, T-2 toxin, HT-2 toxin, Glucoside, Masked mycotoxins, Food processing

## Abstract

Plant-derived phase II metabolites of T-2 toxin (T2) and HT-2 toxin (HT2) were first described in 2011 and further characterized in the following years. Since then, some efforts have been made to understand their biosynthesis, occurrence, toxicity, toxicokinetics, and finally relevance for consumers. Thus, the probably most important question is whether and how these metabolites contribute to toxicity upon hydrolysis either during food processing or the gastrointestinal passage. To answer this question, firstly, knowledge on the correct stereochemistry of T2 and HT2 glucosides is important as this affects hydrolysis and chemical behavior. So far, contradictory results have been published concerning the number and anomericity of occurring glucosides. For this reason, we set up different strategies for the synthesis of mg-amounts of T2, HT2, and T2 triol glucosides in both α and ß configuration. All synthesized glucosides were fully characterized by NMR spectroscopy as well as mass spectrometry and used as references for the analysis of naturally contaminated food samples to validate or invalidate their natural occurrence. Generally, 3-*O*-glucosylation was observed with two anomers of HT2 glucoside being present in contaminated oats. In contrast, only one anomer of T2 glucoside was found. The second aspect of this study addresses the stability of the glucosides during thermal food processing. Oat flour was artificially contaminated with T2 and HT2 glucosides individually and extruded at varying initial moisture content and temperature. All four glucosides appear to be more stable during food extrusion than the parent compounds with the glucosidic bond not being hydrolyzed.

## Introduction

**Fig. 1:**
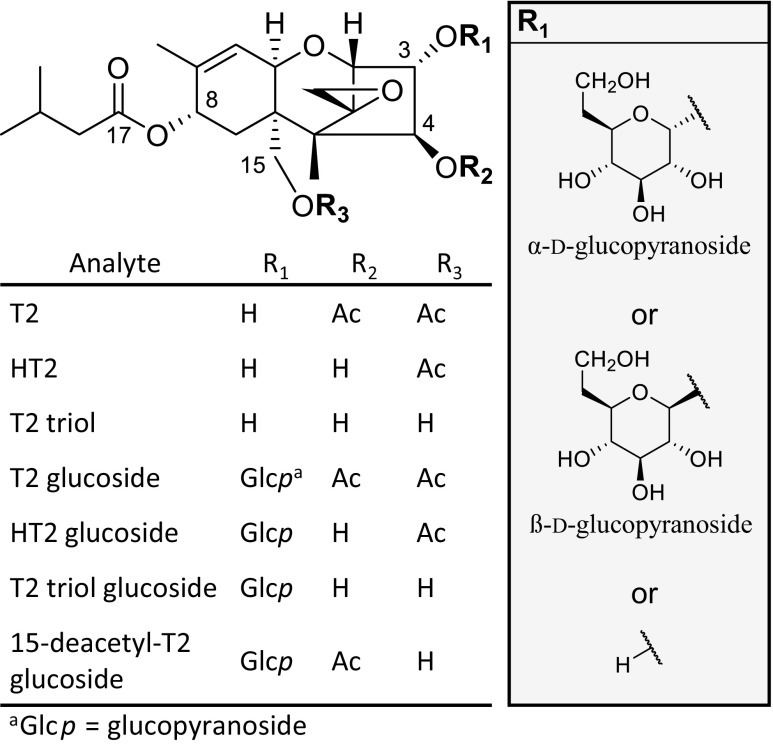
Chemical structures of T-2 toxin (T2), HT-2 toxin (HT2), and T2 triol as well as their respective α or ß configured glucopyranosides (Glc*p*)

The *Fusarium*-toxins T-2 toxin (3α-hydroxy-4ß,15-diacetoxy-8α-(3-methylbutoxy)-12,13-epoxy-trichothec-9-ene, T2, Fig. 1) and HT-2 toxin (3α,4ß-dihydroxy-15-acetoxy-8α-(3-methylbutoxy)-12,13-epoxy-trichothec-9-ene, HT2, Fig. 1) are both classified as type A trichothecenes. Main producers of these toxic secondary metabolites are *Fusarium sporotrichioides*, *F. poae*, and *F. langsethiae* (Thrane et al. 2004). Ingestion of food derived from moldy grain that is contaminated with T2 and HT2 can cause toxic effects in humans and animals mainly by the inhibition of DNA, RNA, and protein synthesis (Ueno 1984; Pettersson 2011).

The general co-occurrence of T2 and HT2 led to a group tolerably daily intake (TDI) of 100 ng/kg body weight/day that was established by the European Food Safety Authority’s Panel on Contaminants in the Food Chain (EFSA 2011) and recently amended to 20 ng/kg body weight/day (EFSA et al. 2017). Neither the European Union nor the USA have established maximum levels for T2 and HT2 (Mazumeder and Sasmal 2001; European Commission 2006). This is attributed to an inchoate state of scientific knowledge regarding the year-to-year variations in toxin occurrence, the influence of agronomic and processing factors, and the presence of modified forms of both toxins in food and feed (European Commission 2013).

Especially, the last aspect has generated attention since the discovery of modified forms of T2 and HT2 in *Fusarium* culture material (Busman et al. 2011) and in food (Lattanzio et al. 2012). These modifications include mainly conjugation of sugars, e.g., glucose as glucopyranosides (Glc*p*) but also conjugates with minor organic acids such as ferulic acid and glutathione are described (Nathanail et al. 2015; Meng-Reiterer et al. 2015, 2016).

Generally, it is assumed that plant-derived phase II metabolites of mycotoxins are less toxic than the parent mycotoxin, as long as the parent toxin is not released upon hydrolysis either during food processing or in the digestive tract (Gratz 2017). Thus, studies investigating the stability of conjugated mycotoxins are of high relevance.

Only two studies analyzed the stability of T2 and HT2 glucosides during food processing (De Angelis et al. 2013; Lattanzio et al. 2015). Both reports revealed that T2 and HT2 glucosides were still detectable after baking (De Angelis et al. 2013) and malting (Lattanzio et al. 2015). However, no final conclusions could be drawn as reference standards for quantification were missing.

In regard to the stability of T2 glucosides upon ingestion, it was shown that T2-3-*O*-α-Glc*p* withstands digestion in the upper gastrointestinal tract in vitro (Gratz et al. 2017) but both α and ß anomers were degraded under simulated human colon microbiota conditions by approx. 60 to 80% (McCormick et al. 2015; Gratz et al. 2017). In broiler chickens, no hydrolysis of orally or intravenous administered T2-3-*O*-α-Glc*p* was observed in the analyzed blood samples (Broekaert et al. 2017). Due to the limitations of these studies, no conclusion can be drawn regarding the stability of T2-Glc*p* and HT2-Glc*p* in humans, demonstrating a need for additional studies in mammals and moreover the need for purified and fully characterized T2-Glc*p* and HT2-Glc*p* standards that can be used in these trials.

There are inconsistent reports on the number of glucoside metabolites of T2 and HT2 occurring in naturally contaminated food and feed. While some sources reported the occurrence of one T2 and one HT2 glucoside (Busman et al. 2011; De Angelis et al. 2013), others noted an additional HT2 glucoside (Lattanzio et al. 2012; Veprikova et al. 2012). Researchers are also divided on the anomericity of T2 glucoside in naturally contaminated materials whether it is in α configuration (McCormick et al. 2015) or not (Meng-Reiterer et al. 2015). In the case of HT2 glucoside, the occurrence of one HT2-3-*O*-ß-glucoside is reported in some cases without presenting characterizing NMR data (Michlmayr et al. 2015b) while other authors described two HT2 glucoside peaks presumably caused by the presence of two different sites for glucosylation at C-3 and C-4 (Lattanzio et al. 2012; Veprikova et al. 2012). In the case of diglucosides of T2 and HT2 toxin, only the HT2 metabolites have been reported by several authors so far (Veprikova et al. 2012; Meng-Reiterer et al. 2015).

Thus, reference standards are urgently needed to clarify the structures of these modified mycotoxins with NMR characterization. In literature, the synthesis of T2-3-*O*-α-Glc*p* starting from T2 using cultures of *Blastobotrys muscicola* converting the aglycone to the Glc*p* has been reported (McCormick et al. 2012). A chemical approach towards an α/ß-mixture of both anomers of T2 glucoside was also described in a consecutive study (McCormick et al. 2015). Glucosylation of HT2 is more complicated since two sites are available for direct glucosylation by plants in α or ß configuration. A biosynthetic approach as used for the synthesis of T2-3-*O*-α-Glc*p* was not successful (McCormick et al. 2012). Instead, ß-glucosides of HT2 could be obtained from experiments using recombinant UDP-glucosyltransferases (UGT) out of *Arabidopsis thaliana* (Poppenberger et al. 2003), rice (Michlmayr et al. 2015a), or barley (Schweiger et al. 2010) expressed in yeast or *Escherichia coli*. Another possible approach is based on exploiting bacterial induced transformations of mycotoxins. With respect to T2, this reactions involve mainly deacetylation to form HT2 and T2 triol (Beeton and Bull 1989) and deepoxydation (Swanson et al. 1987).

In this work, we compare different strategies for the production of T2 and HT2 glucosides with the aim to clarify the structure of the relevant T2 and HT2 monoglucosides by full structure elucidation. One approach towards the synthesis of α-glucosides exploits biotransformation reactions by yeasts and bacteria. For β-glucosides, a chemical approach was tested. To determine the natural occurrence of these compounds, mass spectra and retention times in HPLC were compared with those obtained from naturally contaminated samples. Finally, flour samples artificially spiked with α and ß configured glucosides of T2 and HT2 were subjected to food extrusion to study the stability of the glucosidic bond.

## Materials and methods

### Chemicals and reagents

Chemicals in the highest purity available were purchased from either Sigma-Aldrich (Steinheim, Germany), Carl Roth (Karlsruhe, Germany), or Fisher Scientific (Schwerte, Germany) if not mentioned otherwise. ASTM type 1 water was used at all times unless its non-use is explicitly mentioned, produced with a Purelab Flex 2 system from Veolia Water Technologies (Celle, Germany). T2 and HT2 were produced according to a previously reported protocol (Beyer et al. 2009). A small amount of T2-3-*O*-ß-Glc*p* for analytical purposes was kindly provided by Susan P. McCormick (U.S. Department of Agriculture, Peoria, Il, USA). Whole oat flour and maize grits were purchased from Herrnmühle (Reichelsheim, Germany) and white rice flour was obtained by Ziegler & Co. (Wunsiedel, Germany). Naturally contaminated oat kernels that were used as a reference of naturally occurring T2 and HT2 glucosides were provided by Jens C. Meyer (H. & J. Brüggen, Lübeck, Germany).

### Preparation of T2- and HT2-3-O-α-glucosides by biotransformation

*Streptomyces viridifaciens* DSM 40239 (German Collection of Microorganisms and Cell Cultures, Braunschweig, Germany) stock cultures were stored on glucose yeast malt (G-YM) agar plates at 4 °C. Twenty milliliters G-YM medium in a 50-mL Erlenmeyer flask were inoculated with approx. 0.75 cm^2^ of agar covered with *S. viridifaciens*. Following incubation for 3 days in the dark at 28 °C and 200 rpm on a horizontal shaker, 1 mL of the culture suspension was transferred to a 200-mL Erlenmeyer flask containing 100 mL G-YM medium and incubated again for 2 days in the dark at 28 °C and 200 rpm.

Cultivation and inoculation conditions for *B. muscicola* are based on McCormick et al. (2012). Stock culture suspensions of *B. muscicola* CBS 10338 (Westerdijk Fungal Biodiversity Institute, Utrecht, The Netherlands) were prepared in 50:50 (*v*/*v*) glycerol and filter-sterilized glucose yeast nitrogen base (G-YNB) (Difico) medium and stored at − 80 °C. Prior to usage, *B. muscicola* cultures were grown on yeast malt (YM) agar plates in an incubator for 2 days in the dark at 28 °C. Unlike McCormick et al., the temperature was increased to 28 °C to facilitate bacterial growth in the following. These cultures were transferred to 15 100-mL Erlenmeyer flasks containing 20 mL G-YNB medium with an inoculation loop and grown for 4 days in the dark at 28 °C and 200 rpm. Cultures were centrifuged (10 min, 1000×*g*) and each pellet was resuspended in 80 mL G-YNB medium. One milliliter of the 2-day-old *S. viridifaciens* suspension in G-YM medium was added to every flask.

One hundred microliters of T2 in methanol (76.9 mg/mL, 0.016 mmol) was added to the flasks. One hundred microliters methanol without T2 was added to two of the flasks acting as a growth control. Reaction monitoring was done by HPLC-MS/MS using calculated transitions in selected reaction monitoring mode (SRM). After 14 d of incubation in the dark at 28 °C and 200 rpm, the mixed culture suspensions were centrifuged (10 min, 1000×*g*). The pellets were resuspended in small amounts of water, centrifuged again, and the supernatants were combined. A liquid-liquid extraction using 400 mL *tert*-butyl methyl ether was applied three times to separate the hydrophilic glucosides from the aglycones. The volume of the aqueous layer was reduced on a rotary evaporator to approx. 100 mL and applied for solid phase extraction on a Strata® C18-E phase (10 g/60 mL) (Phenomenex LTD, Aschaffenburg, Germany) to remove residual medium ingredients. The cartridge was equilibrated and loaded at an organic content of 10% methanol, washed with 10–20% methanol, and eluted with 100 mL of 70% methanol. To isolate glucosides in high purity, a preparative HPLC was performed on a ReproSil-Pur 120 C18-AQ column by Dr. Maisch GmbH (Ammerbuch-Entringen, Germany) using a preparative HPLC-system (Jasco Deutschland GmbH, Pfungstadt, Germany) equipped with an UV detector operating at *λ* = 195 nm. Isocratic elution was applied at 28% ACN. Three fractions were isolated and evaporated to dryness yielding 2.8 mg (2.2%) HT2-3-*O*-α-Glc*p*, 3.8 mg (3.0%) 15-deacetyl-T2-3-*O*-α-Glc*p*, and 57 mg (42%) T2-3-*O*-α-Glc*p*.

### Chemical synthesis of T2-, HT2-, and T2 triol-3-O-ß-glucosides

The synthesis of T2-3-*O*-ß-Glc*p* followed the reaction scheme of McCormick et al. (2015) using ethyl 2,3,4,6-tetra-*O*-acetyl-1-thio-ß-d-glucopyranoside (Synthose, Concord, Canada) as starting material. Briefly, the four acetate groups were cleaved and replaced by triisopropylsilyl residues. Upon reaction of the glucose moiety with T2, the triisopropylsilyl ethers were cleaved yielding a mixture of α and ß configured glucosides. Twelve milligrams (13%) of T2-3-*O*-ß-Glc*p* were obtained after a cleanup via preparative HPLC, using the same conditions as applied for the purification of α-glucosides. Comparison of spectroscopic data and HPLC retention time to a reference of T2-3-*O*-ß-Glc*p* and published NMR data confirmed the structure of the ß-glucoside.

Alkaline hydrolysis of T2-3-*O*-ß-Glc*p* to obtain HT2-3-*O*-ß-Glc*p* and further deacetylation products was carried out in four 4-mL glass vials. Twelve milligrams (0.019 mmol) of T2-3-*O*-ß-Glc*p* were dissolved in 0.5 mL methanol, and 125 μL of this solution was added to each vial containing 1 mL 1 m ammonium hydroxide in methanol/water (4:1, *v*/*v*). The glass vials were closed and heated for 40 min at 60 °C with constant stirring. The reaction was stopped by the addition of 100 μL 10 m formic acid, the four vials were combined, and the reaction products isolated via preparative HPLC. Applying the chromatographic conditions described above for the purification of the α-glucosides resulted in three fractions that showed UV absorption at *λ* = 195 nm with corresponding *m*/*z* of 567.2403, 609.2518, and 651.2623. After solvent removal and NMR spectroscopy, these fractions could be identified as T2 triol-3-*O*-ß-Glc*p* (2.0 mg, 19%), HT2-3-*O*-ß-Glc*p* (4.5 mg, 40%), and T2-3-*O*-ß-Glc*p* (4.1 mg, 34%).

### NMR spectroscopy

NMR experiments were performed in *d*_4_-methanol with 0.03% tetramethylsilane (Armar, Döttingen, Switzerland) on an Agilent DD2 600 MHz NMR spectrometer (Agilent Technologies, Waldbronn). Chemical shifts δ are reported as parts per million (ppm) in relation to tetramethylsilane. Proton and carbon spectra and gCOSY, gHMBC, gHSQC, and zTOCSY correlation spectra were recorded. Data processing was done using the Mestrenova software (v. 10.0.0-14381) (Mestrelab Research, Santiago de Compostela, Spain).

### HRMS analysis

Exact mass measurements were done by direct infusion into a LTQ Orbitrap XL mass spectrometer (Thermo Fisher Scientific, Bremen, Germany) equipped with a heated electrospray ionization source. Mass spectra were acquired in positive full scan mode in mass ranges from *m*/*z* 150 to 700 with a resolving power of 30,000. Higher energy collision dissociation (HCD) spectra of the specific *m*/*z* of Na^+^ adducts were recorded with a relative collision energy of 35%. Source conditions were set as follows: capillary temperature, 275 °C; sheath gas flow rate, 5 arbitrary units; source voltage, 4.0 kV; capillary voltage, 40 V; and tube lens voltage, 130 V. Neither sweep gas nor auxiliary gas was applied. For the identification of T2 phase II metabolites HT2-3-*O*-α-Glc*p*, 15-deacetyl-T2-3-*O*-α-Glc*p*, T2-3-*O*-α-Glc*p*, T2 triol-3-*O*-ß-Glc*p*, HT2-3-*O*-ß-Glc*p*, and T2-3-*O*-ß-Glc*p* via exact mass measurements the following data were acquired:

HT2-3-*O*-α-Glc*p*: exact mass (FTMS), *m*/*z* 609.2512 for the [M + Na]^+^ ion (calcd 609.2523 for C_28_H_42_O_13_Na); MS/MS (HCD, 35%) *m*/*z* (%) 245.0631 (6.9), 285.1096 (16), 345.1307 (17), 447.1622 (79), 447.1984 (5.5), 507.1830 (83), 609.2512 (100).

15-deacetyl-T2–3-*O*-α-Glc*p*: exact mass (FTMS), *m*/*z* 609.2517 for the [M + Na]^+^ ion (calcd 609.2523 for C_28_H_42_O_13_Na); MS/MS (HCD, 35%) *m*/*z* (%) 285.1099 (5.2), 345.1310 (14), 417.1521 (9.6), 447.1626 (11), 447.19905 (3.4), 507.1835 (100), 609.2517 (77).

T2-3-*O*-α-Glc*p*: exact mass (FTMS), *m*/*z* 651.2621 for the [M + Na]^+^ ion (calcd 651.2629 for C_30_H_44_O_14_Na); MS/MS (HCD, 35%) *m*/*z* (%) 327.1203 (10), 387.1414 (13), 407.1700 (24), 489.1729 (31), 489.2089 (10), 549.1940 (37), 651.2621 (100).

T2 triol-3-*O*-ß-Glc*p*: exact mass (FTMS), *m*/*z* 567.2403 for the [M + Na]^+^ ion (calcd 567.2417 for C_26_H_40_O_12_Na); MS/MS (HCD, 35%) *m*/*z* (%) 245.0630 (7.7), 405.1878 (0.03), 435.1621 (8.9), 465.1725 (100), 537.2301 (0.9), 567.2403 (64).

HT2-3-*O*-ß-Glc*p*: exact mass (FTMS), *m*/*z* 609.2518 for the [M + Na]^+^ ion (calcd 609.2523 for C_28_H_42_O_13_Na); MS/MS (HCD, 35%) *m*/*z* (%) 245.0634 (8.5), 285.1101 (7.2), 447.1629 (93), 447.1981 (63), 507.1838 (100), 609.2518 (100).

T2-3-*O*-ß-Glc*p*: exact mass (FTMS), *m*/*z* 651.2623 for the [M + Na]^+^ ion (calcd 651.2629 for C_30_H_44_O_14_Na); MS/MS (HCD, 35%) *m*/*z* (%) 327.1206 (6.6), 407.1703 (33), 489.1731 (60), 489.2080 (1.5), 549.1944 (52), 651.2623 (100).

### Food extrusion

A 3:2-mixture of oat and rice flour was used for extrusion experiments. This flour mixture was analyzed for the presence of T2, HT2, and their glucosides prior to extrusion with the method described below. T2 and HT2 were found at negligible levels of 1.1 and 1.9 μg/kg whereas T2 and HT2 glucosides signals remained below the limit of detection. The moisture level of this mixture was analyzed using a standard dry oven method (Matthey and Hanna 1997) and adjusted subsequently to 20 and 30% with tap water. By adding defined amounts of HT2-3-*O*-α-Glc*p*, HT2-3-*O*-ß-Glc*p*, T2-3-*O*-α-Glc*p*, and T2-3-*O*-ß-Glc*p* individually to the tap water used for moistening, the toxin level in 300-g batches of the flour mixtures was adjusted to 250 μg/kg, calculated on a dry weight basis (dwb). Non-fortified batches were prepared for extrusion as a blank reference and to calculate matrix effects and analyte recovery. Each batch was sieved through a 0.8-mm sieve to avoid clumping and to obtain a homogeneous toxin distribution, plastic bagged and stored for 1 h at room temperature for equilibration.

All extrusion cooking experiments were performed on a single-screw stand-alone extruder KE 19/25 (Brabender, Duisburg, Germany). The overall barrel length was 48 cm and the length-to-diameter ratio was 25:1. The engine power was 1.5 kW with a maximum screw torque of 150 Nm. All experiments were done using a 4:1 core progressive screw operating at 150 rpm and a 1:1 feeding screw of the same diameter operating at 30 rpm. The temperatures of the four individual heating zones of the barrel were set to either 70, 100, 100, 120 °C (low temperature conditions) or to 70, 100, 160, 200 °C (high temperature conditions). A round strand die head with 2 mm inner diameter was used. Screw rotation speeds, pressure, temperature in each heating zone, and die temperature were monitored to ensure a stable process quality.

To start the extrusion process, moistened maize grits (25% moisture level) were used. A constant pressure and temperature was awaited before blank flour mixtures were fed to the extruder. Sample collection (approx. 40 g each) was initiated 2 min after that to assure that no maize grits were collected unintentionally.

Three samples were collected for each fortified flour mixture and every moisture and temperature combination and stored at − 20 °C prior to analysis. The extruder was purged with maize grits before another glucoside of T2 or HT2 was fed to the extruder to avoid unwanted carry-over. The moisture level of each extrudate was determined after cooling down, using the drying oven method. Thus, all calculations are based on dry weight.

### Sample preparation

Preparation of all sample material for HPLC-MS/MS analysis was done according to Schmidt et al. (2017). Briefly, 5.0 ± 0.1 g sample were extracted with 20 mL of ACN, H_2_O, and formic acid (79:20:1, *v*/*v*/*v*) using an Ultraturrax® T25 disperser (Ika Werke, Staufen, Germany), followed by centrifugation (10 min, 15,000×*g*) and dilution by a factor of 8 in H_2_O.

To determine the recovery rates of the four mycotoxin glucosides, non-spiked extrudates of each moisture and temperature combination were fortified individually to 25, 125, and 250 μg/kg (dwb) in triplicate by adding the toxins in small amounts of ACN to 5.00 ± 0.05 g sample material. These samples were left open overnight for solvent evaporation and analyzed as described above.

Quantitation was done via matrix-matched calibration curves. Two stock solutions, one containing 1 μg/ml of T2, HT2, T2-3-*O*-α-Glc*p*, and HT2-3-*O*-α-Glc*p* and the other one containing 1 μg/mL of T2, HT2, T2-3-*O*-β-Glc*p*, and HT2-3-*O*-β-Glc*p* were prepared in ACN. From these stock solutions, for every moisture and temperature combination, two individual calibration curves were prepared resulting in a total of eight calibration curves. Therefore, aliquots of the standards were transferred into 1.5-mL glass vials, the solvent evaporated, and the residue dissolved in 125 μL blank matrix extract, followed by 875 μL of water (1:8 dilution). The calibration curves comprised the range from 0.1 to 10 ng/mL (3.2–320 μg/kg) in seven dilution steps. The slope of the matrix-matched calibration curves was used to calculate the sensitivity.

### Liquid chromatography-mass spectrometry analysis

HPLC-MS/MS analysis of all sample extracts and references was done on an Agilent 1260 Infinity series HPLC (Agilent Technologies) coupled to a QTrap® 6500 mass spectrometer with an electrospray ionization (ESI) source (Sciex, Darmstadt, Germany). The Analyst® software (v. 1.6.2, Sciex) was used for data acquisition and processing. The mass spectrometric parameters for all analytes were optimized by syringe pump infusion of the analytes solved in ACN/H_2_O (1:1, *v*/v). MS/MS fragment spectra and enhanced product ion spectra (EPI) of the NH_4_^+^ and the Na^+^ adducts were acquired for comparative purposes.

A Nucleodur® C_18_ Gravity-SB column (100 × 2 mm, 3 μm particle size, Macherey Nagel, Düren, Germany) equipped with a guard column (4 × 2 mm) of the same material was used as a stationary phase. Column oven temperature was set to 40 °C and the thermostatted sample rack was set to 8 °C. The sample injection volume was 5 μL. A binary gradient with methanol (eluent A) and H_2_O (eluent B) at a constant flow rate of 500 μL/min was applied as follows: 0 min, 45% A; 3 min, 45% A; 6 min, 55% A; 11.5 min, 100% A; 12.5 min, 100% A; 12.6 min, 45% A; and 14.0 min, 45% A. The ESI interface was operated in positive ionization mode with the following settings: ionization voltage, 5.5 kV; curtain gas, 35 psi; nebulizer gas, 35 psi; heater gas, 45 psi; source temperature, 450 °C; and entrance potential, 10 V. Declustering potential (DP) and collision energy voltage (CE) were adjusted to each analyte (NH_4_^+^ and Na^+^ adducts) and SRM transition. The sodium adducts gave the highest signal-to-noise ratios (S/N) and were used for quantitation. SRM transitions were: **T2 tetraol**, DP = 100 V, *m*/*z* (CE) 321 → 285 (22 V), 321 → 102 (26 V); **neosolaniol**, DP = 44 V, *m*/*z* (CE) 400 → 245 (15 V), 400 → 215 (21 V); **T2 triol**, DP = 116 v, *m*/*z* (CE) 405 → 303 (22 V), 405 → 125 (23 V); **T2 triol-3-*O*-ß-Glc*p***, DP = 57 V, *m*/*z* (CE) 567 → 465 (37 V), 567 → 435 (40 V), 567 → 245 (41 V); **HT2**, DP = 110 V, *m*/*z* (CE) 447 → 345 (27 V), 447 → 285 (30 V); **HT2-3-*O*-Glc*p***, DP = 210 V, *m*/*z* (CE) 609 → 507 (39 V), 609 → 345 (44 V), 609 → 285 (46 V); **T2**, DP = 145 V, *m*/*z* (CE) 489 → 327 (32 V), 489 → 245 (36 V); **T2-3-*O*-Glc*p***, DP = 150 V, *m*/*z* (CE) 651 → 549 (43 V), 651 → 489 (47 V), 651 → 407 (49 V), 651 → 387 (50 V).

### Analysis of naturally contaminated oat kernels

Naturally contaminated oat kernels and the glucoside references were analyzed with the abovementioned method. All measurements were done in triplicate. References of α- and ß-glucosides were analyzed separately in every case. For confirmation, sample extracts were also fortified with the assigned glucosides, resulting in an increase of peak area and no additional signals.

### Safety precautions

Mycotoxins and mycotoxin stock solutions were handled with special care using safety gloves and protective clothing throughout the experiments. Toxin waste including the liquids as well as single-use tubes and vials was properly disposed.

## Results and discussion

Modified forms of T2 and HT2, especially the *in planta* formed glucosides, are of increasing interest due to their possible health concern. Although they were discovered more than a half decade ago, the structures of the food-relevant glucosides of T2 and HT2 are not fully characterized and some of the reports in literature are conflicting. For this reason, the full set of T2 and HT2 glucosides in α and ß configuration was prepared and characterized.

### Production of T2 and HT2 glucosides

T2-3-*O*-α-Glc*p* was prepared by biotransformation using the yeast *Blastobotrys muscicola* as previously described (McCormick et al. 2012) and confirmed by others (Lattanzio et al. 2015; Broekaert et al. 2017) and resulted in 57 mg of the desired compound.

T2-3-*O*-ß-Glc*p* was also prepared as previously described by McCormick et al. (2015). In this case, a 3 step synthetic approach starting from ethyl 2,3,4,6-tetra-*O*-acetyl-1-thio-ß-d-glucopyranoside was carried out, yielding in total 12.0 mg of the desired product.

For the preparation of HT2-3-*O*-α-Glc*p* and HT2-3-*O*-β-Glc*p*, deacetylation of the respective T2-derivatives was found to be the most suitable approach. So far, deacetylation of T2 has not yet been described for T2-glucosides, but deacetylation of T2 at C-4 to yield HT2 was reported for bacteria of several genera (Ueno et al. 1983; Kiessling et al. 1984; Beeton and Bull 1989). Furthermore, chemical deacetylation of T2 to HT2 and other derivatives has been studied (Beyer et al. 2009). As T2-3-*O*-α-Glc*p* is already formed via biotransformation, the need for purification of this product before an additional biotransformation was questionable. Thus, instead of pre-purification, a combined incubation of T2 with *B. muscicola* and *S. viridifaciens* to prepare T2-3-*O*-α-Glc*p* and HT2-3-*O*-α-Glc*p* in one pot was investigated. Trichothecenes were reported to have no antibacterial activity and to exert no inhibition on bacterial protein synthesis (Ueno et al. 1973). For this reason, the use of bacteria for biotransforming T2 in mg-amounts was possible. Therefore, T2 was incubated with *B. muscicola* and *S. viridifaciens* to produce T2-3-*O*-α-Glc*p* and deacetylate it simultaneously to HT2-3-*O*-α-Glc*p*. The incubation mixture was monitored by daily HPLC-MS/MS measurements, and the results showed that 14 days was the optimum incubation time in terms of T2 disappearance and formation of the corresponding glucosides. Three compounds that appeared to be the major reaction products were isolated in mg-amounts out of the culture medium and identified by NMR and MS as HT2-3-*O*-α-Glc*p*, 15-deacetyl-T2-3-*O*-α-Glc*p*, and T2-3-*O*-α-Glc*p*, respectively (Table 1, Table 2).

**Table 1:** 600-MHz ^1^H NMR data for six mycotoxin glucosides in α and ß configuration recorded in *d*_4_-methanol at 26 °C (chemical shift, multiplicity, (*J* in Hz))

^1^H-NMR shifts												
Analyte												
Position	T-2-3-*O*-α-Glc*p*		HT-2-3-*O*-α-Glc*p*	15-OH-T-2-3-O-α-Glc*p*			T-2-3-*O*-β-Glc*p*		HT-2-3-*O*-β-Glc*p*		T2 triol-3-*O*-β-Glc*p*	
2	3.82	d	3.72	d	3.78	d	3.71	d	3.61	d	3.59	d
	(4.75)		(4.69)		(4.78)		(4.98)		(4.84)		(4.85)	
3	4.33	dd	4.15	dd	4.27	dd	4.47	dd	4.27	dd	4.20	dd
	(4.77, 3.14)		(4.69, 3.13)		(4.79, 3.05)		(4.99, 3.08)		(4.90, 3.19)		(4.89, 3.27)	
4	5.83	d	4.55	d	6.12	d	5.98	d	4.59	d	4.99	d
	(3.16)		(3.18)		(3.04)		(3.10)		(3.22)		(3.25)	
7α	2.36	dd	2.37	dd	2.28	dd	2.38	dd	2.37	dd	2.30	dd
	(15.16, 5.81)		(15.09, 5.77)		(15.20, 5.99)		(15.21, 5.89)		(15.17, 5.79)		(15.24, 6.04)	
7β	1.99	dd	2.01	dd	1.83	dd	1.94	dd	1.99	dd	1.76	dd
	(15.19, 1.56)		(15.17, 1.54)		(15.21, 1.52)		(15.16, 1.55)		(15.23, 1.55)		(15.30, 1.54)	
8	5.33	d	5.32	d	5.28	d	5.33	d	5.31	d	5.27	d
	(5.57)		(5.55)		(5.77)		(5.74)		(5.55)		(5.77)	
10	5.77		5.74	d	5.77	d	5.78	d	5.75	d	5.76	d
	(5.90)		(5.94)		(6.01)		(5.98)		(5.87)		(5.98)	
11	4.44	d	4.31	d	4.48	d	4.39	d	4.22	d	4.33	d
	(5.93)		(5.94)		(5.95)		(6.00)		(5.86)		(5.99)	
13α	3.04	d	2.97	d	3.02	d	3.04	d	2.97	d	2.95	d
	(3.87)		(4.00)		(3.90)		(3.90)		(4.04)		(3.98)	
13β	2.87	d	2.8	d	2.85	d	2.87	d	2.80	d	2.78	d
	(3.93)		(3.99)		(3.93)		(3.90)		(4.00)		(3.98)	
14	0.74	s	0.81	s	0.79	s	0.74	s	0.80	s	0.84	s
15α	4.36	d	4.29	d	3.96	d	4.38	d	4.30	d	3.86	d
	(12.42)		(12.41)		(12.27)		(12.64)		(12.45)		(12.18)	
15β	4.11	d	4.01	d	3.59	d	4.09	d	3.99	d	3.54	d
	(12.46)		(12.41)		(12.29)		(12.44)		(12.43)		(12.18)	
16	1.74	s	1.74	s	1.75	s	1.74	s	1.73	s	1.74	s
18	2.15	m	2.16	m	2.23	m	2.16	m	2.16	m	2.22	m
19	2.06	m	2.08	m	2.07	m	2.07	m	2.07	m	2.08	m
20	0.96	d	0.97	d	0.97	d	0.97	d	0.97	d	0.97	d
	(6.58)		(6.66)		(6.69)		(6.66)		(6.63)		(6.68)	
21	0.95	d	0.96	d	0.96	d	0.96	d	0.96	d	0.97	d
	(6.65)		(6.55)		(6.71)		(6.61)		(6.63)		(6.66)	
CH_3_ (Ac)	2.09, 2.06	s	2.05	s	2.1	s	2.09, 2.06	s	2.05	s		
1’	4.96	d	5.00	d	4.96	d	4.45	d	4.60	d	4.56	d
	(3.75)		(3.77)		(3.76)		(7.73)		(7.76)		(7.82)	
2’	3.43	dd	3.44	dd	3.41	dd	3.25	dd	3.26	dd	3.27	dd
	(9.81, 3.80)		(9.79, 3.80)		(9.82, 3.80)		(9.09, 7.78)		(9.21, 8.08)		(7.92, 8.99)	
3’	3.71	dd	3.72	dd	3.72	dd	3.35	dd	3.38	dd	3.41	dd
	(9.89, 9.02)		(9.67, 8.97)		(9.80, 9.04)		(8.97, 8.97)		(8.99, 8.31)		(9.00, 8.21)	
4’	3.36	dd	3.35	dd	3.35	dd	3.29	dd	3.34	dd	3.37	dd
	(9.88, 9.04)		(9.77, 8.97)		(9.97, 8.97)		(9.02, 8.42)		(8.92, 8.92)		(8.98, 8.71)	
5’	3.58	ddd	3.75	ddd	3.61	ddd	3.21	ddd	3.28	ddd	3.27	m
	(10.05, 4.88, 2.39)		(9.80, 5.53, 2.11)		(10.01, 4.68, 2.31)		(9.64, 5.80, 2.30)		(9.27, 5.27, 2.25)		overlapped	
6’α	3.76	dd	3.85	dd	3.77	dd	3.83	dd	3.84	dd	3.83	dd
	(11.95, 2.43)		(11.56, 2.17)		(12.03, 2.48)		(12.07, 2.27)		(12.05, 2.29)		(12.08, 2.30)	
6’β	3.69	dd	3.69	dd	3.69	dd	3.65	dd	3.68	dd	3.68	dd
	(11.92, 4.88)		(11.49, 5.54)		(11.91, 4.80)		(12.09, 5.76)		(12.05, 5.30)		(12.10, 5.19)

**Table 2:** 151-MHz ^13^C NMR data for six mycotoxin glucosides in α and ß configuration recorded in *d*_4_-methanol at 26 °C. Chemical shift assignment

^13^C-NMR shifts						
Analyte						
Position	T-2-3-*O*-α-Glc*p*	HT-2-3-*O*-α-Glc*p*	15-OH-T-2-3-O-α-Glc*p*	T-2-3-*O*-β-Glc*p*	HT-2-3-*O*-β-Glc*p*	T2 triol-3-*O*-β-Glc*p*
2	77.79	77.90	78.06	80.32	80.44	80.60
3	82.25	85.45	82.86	83.69	87.13	87.81
4	82.05	79.89	82.39	81.02	79.92	79.81
5	50.01	49.81	49.91	49.91	49.87	49.60
6	44.28	43.91	45.68	44.17	43.99	44.63
7	28.52	28.60	28.72	28.64	28.72	29.22
8	69.37	69.62	69.73	69.18	69.58	69.86
9	137.06	137.04	137.01	137.21	137.29	137.32
10	125.17	125.40	125.40	124.90	125.35	125.58
11	68.60	68.72	69.00	68.34	68.68	69.05
12	65.15	65.21	65.40	65.12	65.43	64.10
13	47.76	47.33	47.89	47.72	47.43	47.59
14	7.13	7.52	6.93	6.90	7.41	7.05
15	65.84	65.88	63.75	65.68	65.86	65.81
16	20.42	20.44	20.37	20.29	20.50	20.44
17	173.95	174.05	174.25	173.81	174.06	174.25
18	44.46	44.54	44.56	44.35	44.58	45.32
19	26.89	29.93	26.87	26.78	26.97	26.94
20	22.76	22.77	22.74	22.61	22.75	22.79
21	22.71	22.72	22.73	22.56	20.50	20.44
CH_3_ (Ac)	21.23 20.78	21.21	20.81	21.07	21.21	
				20.65		
C=O (Ac)	172.41	172.28	172.74	172.09	172.17	
	172.09			172.02		
1’	99.54	99.32	99.77	103.61	104.13	104.50
2’	73.37	73.55	73.55	74.63	74.89	75.01
3’	74.70	74.94	74.80	77.91	78.20	78.13
4’	71.45	71.77	71.51	71.29	71.36	71.31
5’	74.42	74.17	74.44	78.17	78.24	78.18
6’	62.22	62.57	62.20	62.47	62.58	62.52

The fact that hardly any HT2 was found in the culture extract suggests that the glucosylation by *B. muscicola* occurs quickly and precedes bacterial deacetylation of T2 by *S. viridifaciens*. The cleanup procedure is simple and time-saving and was comparable with that of the single incubation with *B. muscicola*. However, since some additional minor by-products were produced, it is necessary to carefully verify the structures of the desired metabolites by NMR and MS as described above. The obtained by-products were analyzed by HRMS and they commonly showed minor changes in their sum formula, like for instance the loss of CH_2_ at the isovaleric acid moiety (confirmed in fragmentation experiments), the loss of oxygen, or the addition of C_2_H_2_O. If done properly, this biotransformation approach is a powerful and time-saving tool for the production of mg-amounts of T2 and HT2 glucosides in one single culture flask that does not require complex purification methods.

As T2-3-*O*-β-Glc*p* was already synthesized and purified, a chemical hydrolysis was used to prepare HT2-3-*O*-β-Glc*p*. Furthermore, coupling of HT2 with glucose following the abovementioned protocol was not favored as selectivity directing the pyranose towards position 3 is not given for HT2. Exposure of the prepared T2-3-*O*-ß-Glc*p* for 40 min to a mild hydrolysis by 1 m ammonium hydroxide at 60 °C yielded HT2-3-*O*-ß-Glc*p* and T2 triol-3-*O*-ß-Glc*p* next to the unaltered T2-3-*O*-ß-Glc*p*. The experiment was performed in small scale in advance and monitored via HPLC-MS/MS. This revealed that 40 min is the optimum reaction time as the amount of HT2 glucoside started to decrease with longer reaction time. Upon preparative HPLC cleanup, all products were obtained in mg-amounts sufficient for NMR characterization. No likely products (T2, HT2, T2 triol, neosolaniol, T2 tetraol) were observed after 40 min of hydrolysis, indicating that the glucosidic linkage is stable under these conditions. Increasing the temperature to 60 °C correlated with a shorter reaction time (approx. 90%) compared to the T2 hydrolysis at 30 °C (Beyer et al. 2009). It seems likely that this mild hydrolysis could be applied to the production of α configured glucosides of T2 derivatives.

### NMR characterization of T-2 glucosides

Proton NMR data of the isolated glucosides are reported in Table 1 and carbon NMR shifts in Table 2. The focus of this comparison lays on the glucose moiety and the neighboring atoms of the T2/HT2 molecule and whether signals according to α or ß configuration are shifted in the same way. As reported for T2, α- and ß-glucoside differ in the shifts of the anomeric proton and carbon signals as well as in the *J*_1′-2′_ coupling constant which is increased in case of large axial-axial dihedral angles (β) but is diminished for an equatorial-axial dihedral angle (α) as predicted by the Karplus equation. Comparing the obtained α and ß configured glucosides, we observed the expected signals and coupling constants that are characteristic for their anomericity. The *J*_1′-2′_ coupling constant used to distinguish between α or ß configuration showed *J*_1′-2′_ of 3.71 ± 0.01 Hz for α-glucosides and 7.77 ± 0.05 Hz for ß-glucosides. Furthermore *J*_2′-3′_ was also clearly influenced by the glucose configuration of 9.81 ± 0.02 Hz (α-glucosides) and 9.10 ± 0.11 Hz (ß-glucosides). Additional differences in proton shifts of H-1′, H-2′, H-3′, and H-5′ and carbon shifts for C-2, C-1′, C-3′, and C-5′ were observed. This indicates that a differentiation between α and ß configuration cannot only be made by the *J*_1′-2′_ coupling constant but also by chemical shifts in the respective proton or carbon spectrum.

### Mass spectrometric characterization and HPLC separation of T-2 glucosides

The method used for simultaneous detection of T2 and HT2 and modified mycotoxins is usually HPLC-MS/MS (Bernhardt et al. 2016). The synthesized T2 and HT2 glucosides were used to investigate if the different configuration results in distinguishable chromatographic behavior as well as different MS/MS fragmentation patterns. The latter aspect was studied via direct infusion of a reference solution to a triple quadrupole mass spectrometer with a linear ion trap and to a FTMS.

FTMS fragmentation spectra did not allow us to distinguish between both configurations because no consistent fragmentation pattern was observed. The loss of isovaleric acid (*m*/*z* 102.0681) was one of the most intense transitions in most cases. The loss of the glucose moiety (*m*/*z* 162.0528) was observed for all glucosides but appeared less prominent than the loss of C_7_H_14_O_4_ (probably isovaleric acid and acetic acid or isovaleric acid and two units of formaldehyde, *m*/*z* 162.0892).

**Fig. 2:**
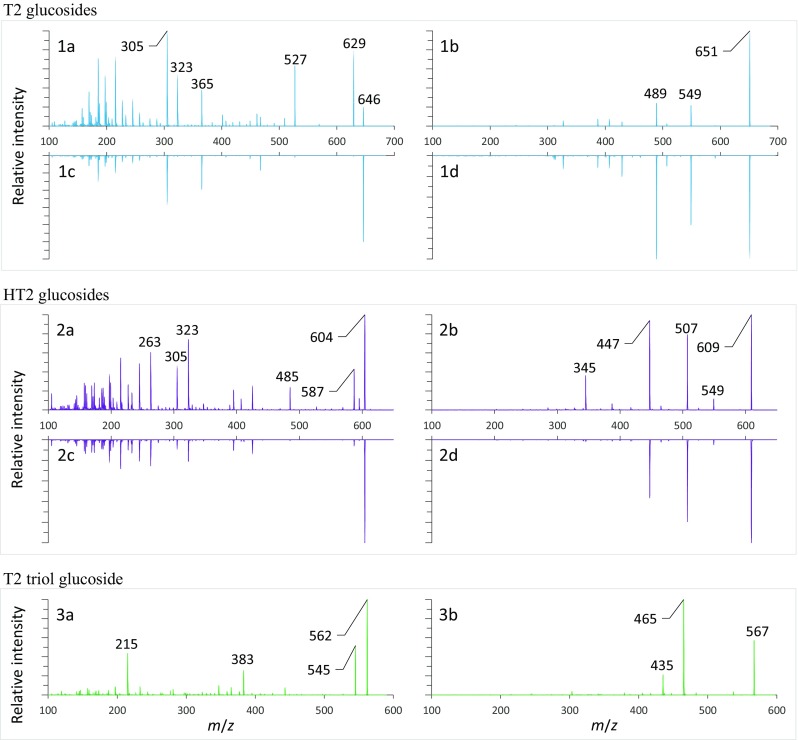
EPI spectra of NH_4_^+^ and Na^+^ adducts of glucosides isolated in this study. NH_4_^+^ adducts are displayed on the left, Na^+^ adducts on the right. All NH_4_^+^ and all Na^+^ adducts were fragmented at 15 and 40 V, respectively. The spectra are shown in the following order: T2-3-*O*-α-Glc*p*, 1a, 1b; T2-3-*O*-ß-Glc*p*, 1c, 1d; HT2-3-*O*-α-Glc*p*, 2a, 2b; HT2-3-*O*-ß-Glc*p*, 2c, 2d; and T2 triol-3-*O*-ß-Glc*p*, 3a, 3b

Fragmentation experiments were also carried out on a triple quadrupole mass spectrometer using the most frequently reported NH_4_^+^ and Na^+^ adducts of T2 and HT2. With only minor exceptions, the optimum CE of all major fragments from one ion species were in the same range, namely 35–50 V for Na^+^ adducts and 10–30 V for NH_4_^+^ adducts. Consequently, we recorded EPI spectra of the glucosides with the same, comparable CE (Na^+^ adducts, 40 V; NH_4_^+^ adducts, 15 V). All spectra are shown in Fig. 2. Comparing the spectra of α and ß anomers of T2 and HT2 glucoside, we observed similar fragmentation patterns but different ratios in the fragment intensities for sodium and ammonium adducts allowing to distinguish between α and ß form. The *m*/*z* 527 and *m*/*z* 485 fragments of ammonium adducts of T2 and HT2 glucosides that are formed by the loss of ammonia and isovaleric acid enable a rough distinction between α and ß form. Regarding T2 glucoside, this was first described by McCormick et al. (2015) but other authors also observed the ion transition *m*/*z* 646 → 527 (Nakagawa et al. 2012; Veprikova et al. 2012; Lattanzio et al. 2013).

Furthermore, for both ion species of all six glucosides, the CE was optimized individually for every relevant product ion. On average, the glucoside anomericity influenced the CE optimum by 5% or less. In this way, single SRM transitions can be applied for the analysis of both anomers to reduce the cycle time or extend the dwell time for improving the S/N ratio.

**Fig. 3:**
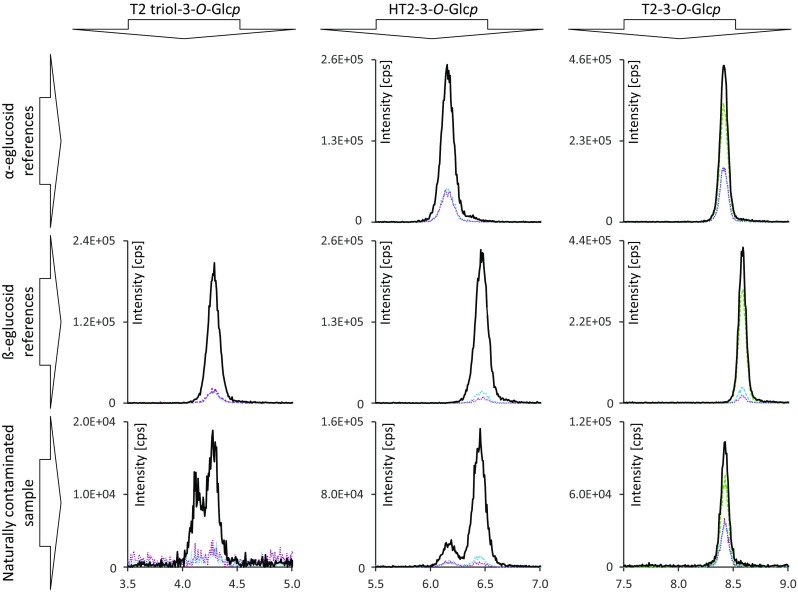
Sections of HPLC-MS/MS chromatograms recorded in SRM mode. The bottom row shows SRM transitions of T2 triol, HT2 and T2 glucosides from left to right in the order of elution. In the top row, peaks corresponding to HT2-3-*O*-α-Glc*p* and T2-3-*O*-α-Glc*p* are displayed. The middle row shows peaks corresponding to T2 triol-3-*O*-ß-Glc*p*, HT2-3-*O*-ß-Glc*p* and T2-3-*O*-ß-Glc*p*. Retention time and SRM transition intensities allow to distinguish between α and ß configuration

In the next step, the optimized MS parameters were applied to develop a chromatographic separation. In the case of T2-glucoside as well as HT2 glucoside, it was possible to separate the respective α- and β-forms chromatographically. Using the specified HPLC-column, in the case of HT2 and T2 glucoside, the α anomer eluted before the ß anomer. Interestingly, and in good accordance with Fig. 2, only the earlier eluting HT2-3-α-Glc*p* gave a signal for the specific SRM transition *m/z* 604 → 485 while HT2-3-β-Glc*p* was not visible for this SRM. The application of the developed method to naturally contaminated samples is shown in Fig. 3. The depicted sections of HPLC-MS/MS chromatograms of T2 triol, HT2, and T2 glucosides of the respective references and a naturally contaminated sample allow a direct assignment. As SRM transitions were recorded at the same CE of the more intense Na^+^ adducts of both anomers, differing SRM ratios additionally allow to distinguish between both forms. The ratios of the SRM transitions monitored in this study are shown in Table 3. In the case of HT2 glucosides, the distinction is enabled by the ratios of the SRM transition *m*/*z* 609 → 285 and *m*/*z* 609 → 345 to the most intense SRM transition *m*/*z* 609 → 507. The respective SRM ratios are 4.7 ± 0.03 and 4.9 ± 0.18 for the α-glucosides and 14.1 ± 0.46 and 32.8 ± 0.69 for the ß-glucosides. A comparison of the signal intensities of the SRM transitions *m*/*z* 651 → 387 and *m*/*z* 651 → 327 to *m*/*z* 651 → 489 for T2 α- and ß-glucoside resulted in ratios of 2.8 ± 0.03 and 2.8 ± 0.18 for the α-glucosides and 22.9 ± 0.70 and 9.6 ± 0.13 for the ß-glucosides. Summing up, we can conclude that the different anomeric forms of T2 and HT2 glucosides can be distinguished chromatographically and by their respective SRM transition ratios.

**Table 3:** Comparison of SRM ratios of aglycones and glucosides that were available as reference compounds to the ratios acquired in naturally contaminated oats

		Ratio of the SRM transitions	
Analyte	*m*/*z*	Reference compounds	Naturally contaminated sample
T2 triol	405 → 303 / 405 → 125	13.0	11.9
T2 triol-3-*O*-α-Glc*p*	567 → 465 / 567 → 435	12.3	13.1
	567 → 465 / 567 → 245	10.0	10.1
HT2	447 → 345 / 447 → 285	1.8	1.8
HT2-3-*O*-α-Glc*p*	609 → 507 / 609 → 447	1.0	1.2
	609 → 507 / 609 → 285	4.7	4.6
	609 → 507 / 609 → 345	4.9	4.7
HT2-3-*O*-ß-Glc*p*	609 → 507 / 609 → 447	1.0	1.2
	609 → 507 / 609 → 285	14.1	13.5
	609 → 507 / 609 → 345	32.8	29.5
T2	489 → 245 / 489 → 327	1.4	1.3
T2-3-*O*-α-Glc*p*	651 → 489 / 651 → 549	1.3	1.4
	651 → 489 / 651 → 407	2.2	2.2
	651 → 489 / 651 → 387	2.8	2.9
	651 → 489 / 651 → 327	2.8	2.7
T2-3-*O*-ß-Glc*p*	651 → 489 / 651 → 549	1.4	n.d.
	651 → 489 / 651 → 407	2.7	n.d.
	651 → 489 / 651 → 387	22.9	n.d.
	651 → 489 / 651 → 327	9.6	n.d.

### Natural occurrence

In order to clarify the anomericity of the naturally occurring glucosides, we compared HPLC elution behavior and the ratio of the SRM transitions of the isolated references to a naturally contaminated oat sample. The respective chromatograms are displayed in Fig. 3. It is noteworthy that in chromatograms of a naturally contaminated sample, two peaks were observed for T2 triol and HT2 glucosides, while T2 glucoside only showed one peak. A comparison of the retention times with the glucoside reference compounds showed that T2-3-*O*-α-Glc*p*, HT2-3-*O*-α-Glc*p*, HT2-3-*O*-ß-Glc*p*, and T2 triol-3-*O*-ß-Glc*p* naturally occur in contaminated oats. The SRM transition ratios were also compared and confirm this conclusion. In the case of the T2 triol glucoside double peak, the latter peak matched our reference in ß configuration. A comparison of the elution behavior of HT2 and T2 glucosides indicates the presence of a naturally occurring T2 triol-3-*O*-α-Glc*p* but as no reference was at hand this could not be confirmed. No peak matching the retention time and the SRM transition ratio of 15-deacetyl-T2-3-*O*-α-Glc*p* was detected in contaminated oats.

In the case of T2 glucoside, our results are in good accordance to McCormick et al. (2015) but contradict the findings of Meng-Reiterer et al. (2015) who reported a retention time mismatch for T2-3-*O*-α-Glc*p* with T2 glucoside occurring in barley. The occurrence of HT2-3-*O*-ß-Glc*p* has already been reported but the authors did not present characterizing NMR data to confirm this finding and no second peak for HT2 glucoside was described (Meng-Reiterer et al. 2015). The absence of ß configured T2 glucoside indicates that HT2-3-*O*-ß-Glc*p* is formed subsequently to C-4 deacetylation of T2. Prior to this, ß configured glucosides were expected for T2, HT2, and T2 triol because ß-linked glucosides of xenobiotics are more commonly found (Bowles et al. 2005) and have been described for the trichothecene deoxynivalenol (Sewald et al. 1992). Therefore, it is most likely that T2 and HT2 are conjugated by different glycosyltransferases favoring different glucoside configuration.

### Thermal stability of T2 and HT2 glucosides during extrusion cooking

The last aspect of this work addressed the stability of T2 and HT2 glucosides during thermal food processing. Extrusion cooking was chosen as this process provides high temperatures and pressure as well as friction. Though T2-3-*O*-ß-Glc*p* was not detected in food, the stability of this glucoside was also investigated in order to compare it with the naturally occurring glucosides. The T2 and HT2 glucosides were quantified using matrix-matched calibration functions. Furthermore, the determined concentrations were corrected for their recovery rates which were for T2-3-*O*-α-Glc*p*, 119 ± 4.8%; T2-3-*O*-ß-Glc*p*, 113 ± 6.9%; HT2-3-*O*-α-Glc*p*, 106 ± 4.6%; and HT2-3-*O*-ß-Glc*p*, 116 ± 4.7%.

**Fig. 4:**
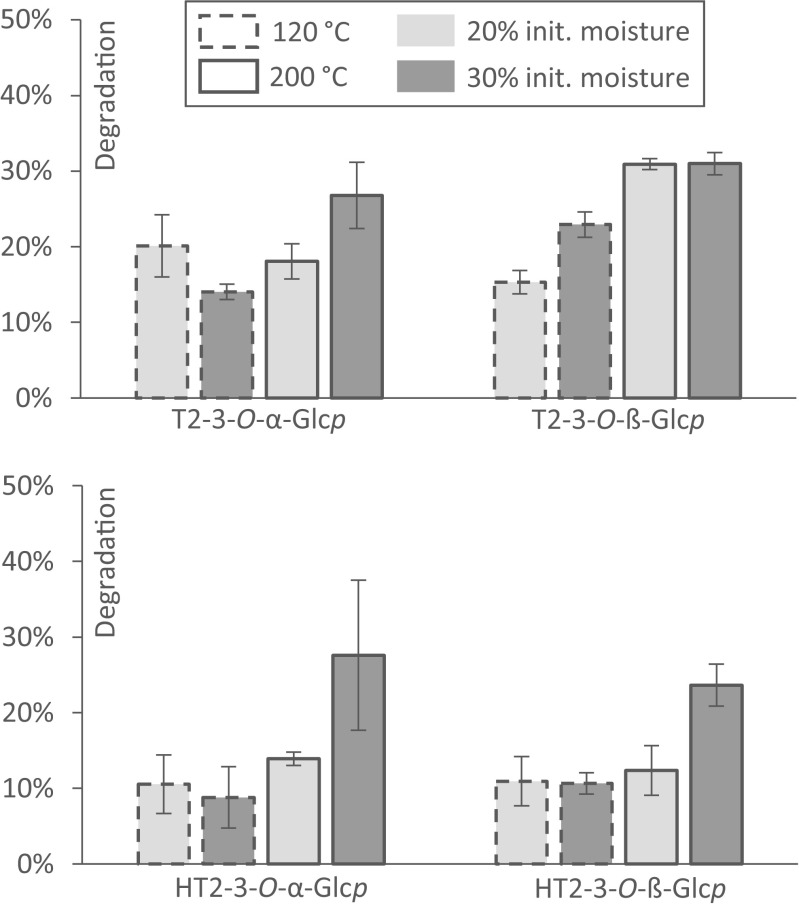
Degradation of T2 and HT2 glucosides during food extrusion. Mixed oat and rice flours were artificially contaminated with the individual analytes and extruded at varying initial moisture level and temperature. Degradation rates were calculated considering the changes in moisture level and analyte recovery

Extrusion cooking experiments were carried out using a 4:1 compression screw and a round strand die with a diameter of 2 mm. The initial moisture level was adjusted to 20 and 30% and both mixtures were extruded at a temperature of either 120 or 200 °C at the die head. The thermal degradation of T2 and HT2 glucosides was evaluated and compared to the degradation rates of T2 and HT2 reported previously on the same extruder using the same settings (Schmidt et al. 2017). The thermal degradation is displayed in Fig. 4 with the variations in initial moisture level and temperature. In most experiments, an increase of the temperature was followed by an increase of the degradation rate. The average degradation rate over all experiments was 20 ± 3.0% for T2-3-*O*-α-Glc*p* and was slightly higher, 25 ± 1.4%, for T2-3-*O*-ß-Glc*p*. As can be seen from Fig. 4, changes in the moisture level and the temperature effected the degradation rates of the T2 glucosides differently, while it showed no change on T2-3-*O*-ß-Glc*p* at 200 °C. The thermal degradation rates of HT2 glucosides were found to be lower than those of T2 glucosides. The average degradation in all experiments was 14 ± 2.8% for HT2-3-*O*-α-Glc*p* and 14 ± 2.7% for HT2-3-*O*-ß-Glc*p*. A change in the temperature from 120 to 200 °C resulted in increased degradation rates of HT2 glucosides, especially at a higher water content (Fig. 4).

The degradation of the four glucosides was generally lower than the degradation of their respective aglycones under the same conditions as recently described by Schmidt et al. with a maximum degradation of T2 by 59 ± 1.5% and of HT2 by 47 ± 0.5% (2017). This was in contrast to our expectation that an additional glucosidic bond that might be cleaved inside the molecule presumably enhances the potential of chemical hydrolysis. We investigated if the loss of glucosides leads to the formation of the respective aglycones by analyzing the extrudates for the presence of T2, HT2, T2 triol, neosolaniol, and T2 tetraol. Though a cleavage of the glucosidic bond would be a plausible explanation for the loss of the glucosides, the aglycone signals remained on the same level as the blank reference. This demonstrated clearly that no T2 or HT2 is released from T2 or HT2 glucoside during extrusion cooking of oats. The question whether and how these glucosides are cleaved during processing that involves fermentation steps (e.g., bread baking, malting) was not addressed in this study and needs to be further investigated in future work.

The unavailability of plant-derived phase II metabolites of mycotoxins often hampers their investigation in terms of occurrence, analytical determination, toxicity, and toxicokinetics. In this work, we report two efficient synthesis strategies for the production of relevant glucosides of T2 and HT2. Characterizing data on six different glucosides of T2 and T2 derivatives are reported in order to contribute to a better understanding of the phase II metabolism of these mycotoxins in plants. A first investigation on their thermal stability during food extrusion revealed that the majority withstands heat, pressure, and shearing and might therefore be present in the final food product. These findings clearly indicate that modified T2 and HT2 should be considered in the toxicity evaluation and human health risk assessment of T2 and HT2.
